# Relaxation processes in hybrid organic-inorganic polymer nanosystems polymerized *in situ*

**DOI:** 10.1186/1556-276X-9-217

**Published:** 2014-05-07

**Authors:** Maksym Iurzhenko, Yevgen Mamunya, Gisele Boiteux, Gerard Seytre, Erisela Nikaj, Olivier Gain, Eugene Lebedev, Svetlana Ishchenko

**Affiliations:** 1 Institute of Macromolecular Chemistry of the National Academy of Sciences of Ukraine, Kyiv 02160, Ukraine; 2 Center for Thermophysical Investigations and Analysis of the NAS of Ukraine, Kyiv 02160, Ukraine; 3 Université de Lyon, Université Lyon 1, Ingénierie des Matériaux Polymères, UMR CNRS 5223, IMP@LYON1, Lyon 69622, France

**Keywords:** Hybrid polymers, Organic-inorganic nanosystems, Joint polymerization, Interpenetrating networks, Relaxation phenomena

## Abstract

The relaxation processes of hybrid organic-inorganic polymer nanosystems (OIS) synthesized by joint polymerization of organic and inorganic components were studied using methods of differential scanning calorimetry (DSC), dynamic mechanical thermal analysis (DMTA), and broadband dielectric relaxation spectroscopy (DRS). The organic component was a mixture of two products: high-molecular-weight macrodiisocyanate (MDI) with low reactivity and low-molecular-weight isocyanate-containing modifier poly(isocyanate) (PIC) with high reactivity. Sodium silicate (SS) was used as inorganic component. The structures of the OIS obtained were in the form of hybrids with covalently connected building blocks and interpenetrating networks: weakly cross-linked network MDI/SS and highly cross-linked network PIC/SS. Depending on the MDI/PIC ratio, one of the networks was prevailing and created a continuous structure with domains of second network.

**PACS:**

61.25.hk; 82.35.Lr; 64.70.pj

## Background

Hybrid organic-inorganic polymer nanosystems (OIS) were considered by many researchers as very interesting and perspective materials due to possibility to combine chemically bonded organic and inorganic blocks in one structure and, therefore, to synthesize compositions with their common properties, thus obtaining materials with specific characteristics [1,2]. OIS represent as perspective industrial materials, such as solid polymer electrolytes and membranes for fuel cells [3,4] (due to the presence of ionic conductivity) and coatings (because of their high chemical, radiation resistance and thermal stability [5-7]).

In general, the investigation of the structure/properties relationships is a major aim of Materials Science [8-10]. Many efforts are applied to the complex investigations of a relaxation behavior of various materials because of ability to obtain the information of these relationships.

The mostly well-known method of synthesis of hybrid organic-inorganic systems is the sol-gel process that is highly effective for synthesis of tailored organic-inorganic systems [1-3,11]. However, this multi-step method involves rather complicated processes. In contrast to the sol-gel method, the method of joint polymerization is very attractive from a technological point of view [10-12]. The major concept of this original method of the OIS synthesis is a polymerization of OIS in a reactive mixture of liquid organic and inorganic oligomers, which have free reactive groups in their molecular structure. Varying organic and inorganic oligomers allows obtaining of the final product, OIS, with a wide range of physical-chemical characteristics.

Previously, we have reported that OIS synthesized by joint polymerization of various organic oligomers and sodium silicate (inorganic component) have different properties, depending on the formed hybrid structures [13-17]. Such OIS have also a high level of ionic conductivity in a wide temperature range due to an ionomeric polymer matrix (high-molecular-weight polyurethane) and presence of a number of the charge carriers, mainly sodium cations Na^+^, in a mineral phase. That makes these OIS perspective polymer materials for solid electrolytes and membranes for fuel cells. Whereas complex electrophysical properties (electric, dielectric and important mechanical characteristics) in respect to structural organization of OIS have not been yet studied, so such relationship by OIS's relaxation behavior is established in the present work.

## Methods

### Materials and processing

Organic component of OIS consists of two isocyanate-containing products:

– urethane oligomer-macrodiisocyanate (MDI) with *Mw* = 4,500 that contains two free reactive NCO groups. MDI was synthesized on the base of 2,4-toluene diisocyanate and oligooxypropyleneglycol with *Mw* = 2,100.

– low-molecular-weight isocyanate-containing modifier poly(isocyanate) (PIC) with *Mw* = 450 and three free reactive NCO groups. PIC was based on a composition 50/50 of diphenylmethandiisocyanate (*Mw* = 250)/isocyanate isomers. PIC of type D was used.

Inorganic component was sodium silicate (SS) existing in the form of oligomer in water solution with the general formula

$$a{\text{Na}}_{2}O\times b{\text{SiO}}_{2}\times cH_{2}O,$$

where *b*/*a* is silicate module. Industrial sodium silicate with characteristics defined by the national standard GOST 13078-81 was used. The value of *b*/*a* is equal to 2.8, and the density is 1.45 g/cm^3^. The detailed characteristics of the products were given in [10].

OIS were synthesized *in situ* in a reactive mixture of organic and inorganic oligomers; the reactions of synthesis were described in [11,12]. Weight ratio of MDI/PIC was varied in the range from 0/100 to 100/0 that gave the opportunity to change the reactivity of the organic component. The ratio of the organic/inorganic components (MDI + PIC)/SS equaled to 70/30 for all hybrid compositions. The reactive mixtures were placed into Teflon moulds (Wilmington, DE, USA) where the OIS curing passed during 24 h at room temperature (*T* = 22°C ± 1°C).

### Equipment and measurements

The differential scanning calorimetry investigations (DSC) were carried out using TA Instruments 2920 MDSC V2.6A (New Castle, DE, USA) in helium and air atmosphere Q50 with cooling/heating rate of 10°C/min in the temperature range from −100°C to +400°C, depending on the thermal stability of OIS that was previously studied at TA Instruments TGA Q50.

The dynamic mechanical thermal analysis (DMTA) was performed using TA Instruments DMA 2980 (New Castle, DE, USA) in the single cantilever mode. The frequency range was taken from 1 to 30 Hz, the amplitude of oscillation was chosen at 20 ± 0.001 μm and the temperature interval was from −100°С to +400°С ± 0.1°С with a heating rate of 3°С ± 0.1°С/min. The OIS samples were in the form of blade with the following dimensions: height was *h* = 1 ± 0.01 mm, width *d* = 6 ± 0.01 mm and length *l* = 40 ± 0.01 mm.

The data of DMTA and DSC measurements were analyzed using the TA Instruments Universal Analysis 2000 ver. 3.9A. The dielectric relaxation spectroscopy (DRS) methods allow studying of the dielectric relaxation phenomena of OIS. The DRS spectra were obtained by Novocontrol Alpha High-Resolution Dielectric Analyzer with Novocontrol Quatro Cryosystem (Montabaur, Germany) equipped with two-electrode scheme. The frequency range was 10^−2^ to 10^7^ Hz, the temperature interval was from −100°С to +400°С ± 0.01°С, and the cooling/heating rate equaled to 3°C/min. The data was analyzed using Novocontrol WinDETA ver 3.8 and Novocontrol WinFIT ver 2.8.

## Results and discussion

The reactivity of the organic component is a relative parameter that is calculated from several chemical characteristics of products [18,19]. The length of molecular chains (molecular weight Mw) and number of reactive groups in the products are the major characteristics. The mobility of molecular chains of products is neglected in this case. Therefore, in the first approximation, the reactivity of the organic component can be calculated using Equation 1:

(1) $$R=\frac{x\cdot Mw_{\text{react}}}{Mw_{\text{comp}}},$$

where *R* is the reactivity of a component, *x* is the number of reactive groups, *Mw*_react_ is the molecular weight of reactive groups, and *Mw*_comp_ is the molecular weight of a component.

For multi-component system, the reactivity is determined by additive contributions of components. In this case, Equation 1 takes the following form:

(2) $$R=\sum_{i=1}^{n}m_{i}\cdot \frac{x\cdot Mw_{\text{react}}}{Mw_{i\;\text{comp}}},$$

where *m*_*i*_ is the content of the *i* component, *x*_*i*_ is the number of reactive groups in the *i* component, *Mw*_react_ is the molecular weight of the reactive groups, and *Mw*_*i*comp_ is the molecular weight of the *i* component. Equation 2 is valid if the reactive groups of all the components have an identical chemical structure.

In our case, Equation 2 takes the following form:

(3) $$R=m_{\text{MDI}}\cdot \frac{x_{\text{MDI}}\cdot Mw_{\text{NCO}}}{Mw_{\text{MDI}}}+m_{\text{PIC}}\cdot \frac{x_{\text{PIC}}\cdot Mw_{\text{NCO}}}{Mw_{\text{PIC}}},$$

where *m*_MDI_ and *m*_PIC_ are the contents of MDI and PIC, *x*_MDI_ = 2 and *x*_PIC_ = 3 are the numbers of the NCO groups in MDI and PIC, *Mw*_NCO_ is the molecular weight of the NCO group, and *Mw*_MDI_ and *Mw*_PIC_ are the molecular weights of MDI and PIC, respectively. The compositions and reactivity of the organic component of OIS are shown in Table 1.

**Table 1:** Reactivity and compositions of the organic component of OIS

**Reactivity (*R*)**	**MDI (%)**	**PIC (%)**
0.04	100	0
0.1	80	20
0.14	65	35
0.16	58	42
0.18	50	50
0.22	35	65
0.26	20	80
0.32	0	100

## DSC results

**Figure 1:**
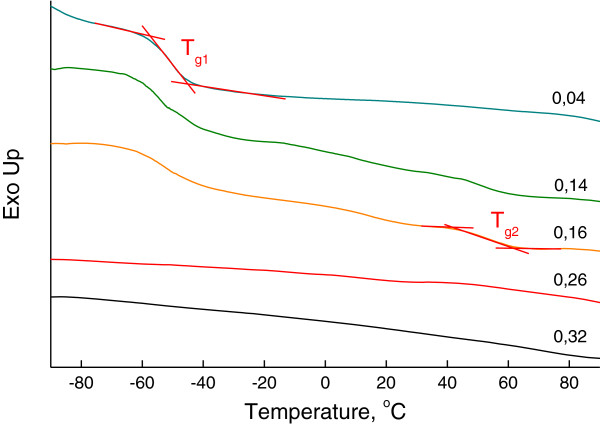
**DSC curves of OIS with different organic component reactivities *R*. ***R* is varied from 0.04 to 0.32.

Figure 1 shows the DSC thermograms of OIS with different organic component reactivities. One can see the presence of several endothermic processes on the thermograms, which confirm the existence of different structural formations in OIS bulk and correspond to their glass transition temperatures. The temperatures of the glass transitions are shown in Table 2. For OIS with reactivity *R* = 0.04, in which the organic component consists of only high-molecular-weight MDI, one glass transition process *T*_g1_ near −50°C can be found and corresponds to elastic hybrid organic-inorganic network MDI/SS that was formed in reactions between the NCO groups of the MDI and OH groups of SS.

**Table 2:** DSC studies: temperatures of the relaxation processes

**Compositions**			**Glass transition temperatures**	
**Reactivity (*R*)**	**MDI (%)**	**PIC (%)**	***T***_**g1 **_**(°C)**	***T***_**g2 **_**(°C)**
0.04	100	0	−50	-
0.1	80	20	−48	39
0.14	65	35	−53	54
0.16	58	42	−58	55
0.18	50	50	−63	59
0.22	35	65	−70	67
0.26	20	80	−76	74

Compositions and glass transition temperatures of OIS obtained from DSC investigations, depending on the reactivity *R* of the organic component of OIS.

The increase of the organic component reactivity *R* by adding PIC in the reactive mixture leads to the appearance of the second glass transition process *T*_g2_ near 40°C. Thus, it can be referred to the more rigid hybrid organic-inorganic network PIC/SS that is formed in reactions between the NCO groups of PIC and the OH groups of SS. Further increase of *R* shifts *T*_g1_ to lower temperatures due to the presence of a low-molecular-weight product that appeared during polymerization and plays the role of plasticizer for elastic network MDI/SS. At the same time, the rise of *T*_g2_ is observed since the plasticizing effect is weak as compared with a strong impact of growing and cross-linking of rigid hybrid network PIC/SS.

## DMTA results

**Figure 2:**
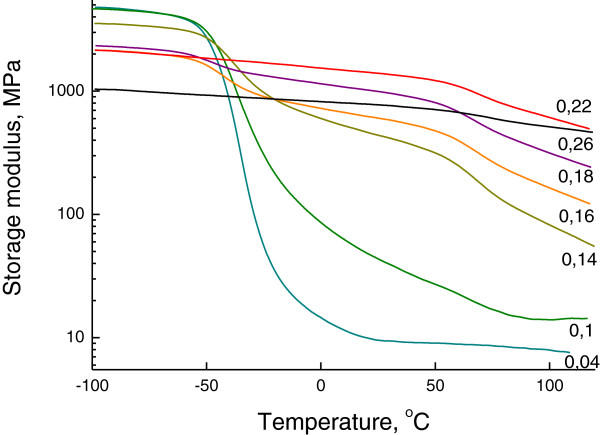
**Storage modulus dependencies of OIS on the reactivity *R *of the organic component of OIS.** Storage modulus curves were obtained by DMTA at frequency *ω* = 1 Hz.

**Figure 3:**
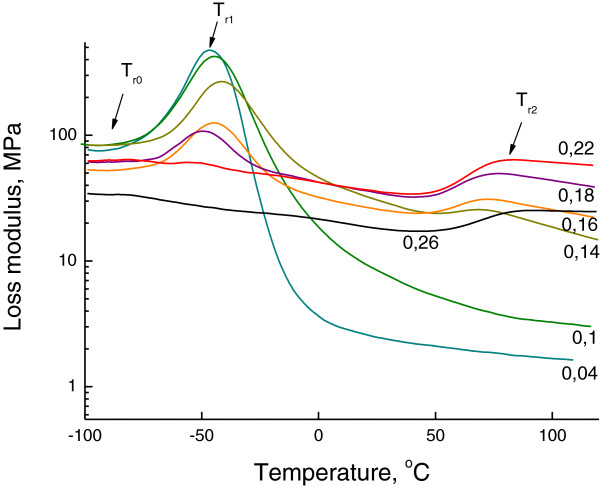
**Loss modulus dependencies of OIS on the reactivity *R *of the organic component of OIS.** The loss modulus curves were obtained by DMTA at frequency *ω* = 1 Hz. Three relaxation processes, namely, at −90°C (*T*_r0_), −50°C (*T*_r1_) and 70°C (*T*_r2_) are pointed on the plot.

The DMTA results show the presence of two (Figure 2) and three (Figure 3) relaxation processes, depending on the composition of OIS. The temperatures of these relaxation processes are noted in Table 3. The relaxation temperatures *T*_r1_ and *T*_r2_ relate to the glass transition temperatures *T*_g1_ and *T*_g2_ and correspond to the hybrid networks MDI/SS and PIC/SS, respectively. A good correlation between values and shifts of relaxation temperatures (DMTA results) and glass transition temperatures (DSC results) is revealed. The third weak relaxation process *T*_r0_ near −90°C (Figure 3) corresponds to the relaxation of a low-molecular-weight product that plays the role of plasticizer for hybrid networks. The rise of *R* leads to the increase of a low-molecular-weight product in OIS bulk and, correspondingly, to the increase of its relaxation temperature and plasticizing effect. The lessening of intensities of defrosting of both hybrid networks due to the increase of content and cross-linking of rigid hybrid network PIC/SS with the rise of *R* is also observed (Figures 2 and 3).

**Table 3:** DMTA studies: temperatures of the relaxation processes

**Compositions**			**Relaxation temperatures (*ω*** **= 1 Hz)**		
**Reactivity (*R*)**	**MDI (%)**	**PIC (%)**	***T***_**r0 **_**(°C)**	***T***_**r1 **_**(°C)**	***T***_**r2 **_**(°C)**
0.04	100	0	−94	−43	-
0.06	90	10	−92	−42	-
0.1	80	20	−89	−39	56
0.14	65	35	−79	−39	64
0.16	58	42	−76	−43	67
0.18	50	50	−73	−46	76
0.22	35	65	−71	−52	82
0.26	20	80	−69	−74	86

Compositions and glass transition temperatures of OIS obtained from DMTA investigations at frequency *ω* = 1 Hz, depending on the reactivity *R* of the organic component of OIS.

## DRS results

**Figure 4:**
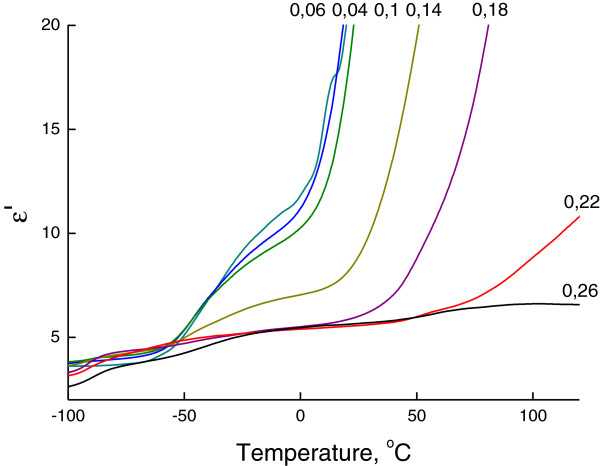
**Permittivity dependencies of OIS on the reactivity *R *of the organic component of OIS.** Permittivity curves were obtained by DRS at frequency *ω* = 1 Hz.

**Figure 5:**
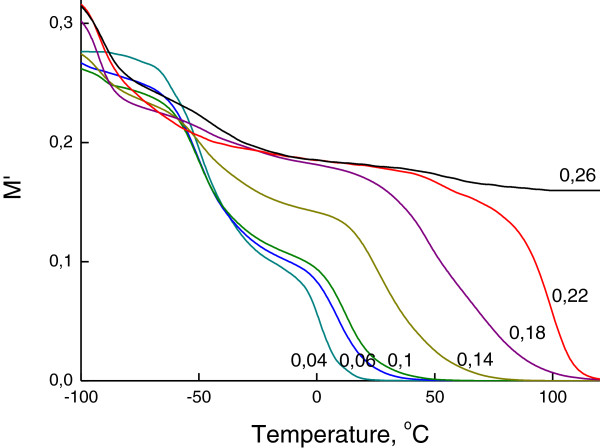
**Dependencies of electrical modulus *M*′ of OIS on the reactivity *R *of the organic component of OIS.** Curves of electrical modulus were obtained by DRS at frequency *ω* = 1 Hz.

**Figure 6:**
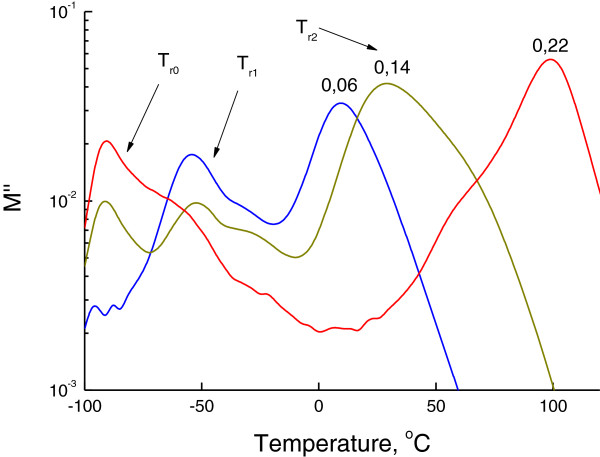
**Dependencies of electrical losses *M*″ of OIS on the reactivity *R *of the organic component of OIS.** Curves of electrical modulus were obtained by DRS at frequency *ω* = 1 Hz. Three relaxation processes, namely, at −90°C (*T*_r0_), −50°C (*T*_r1_) and near 50°C (*T*_r2_) are pointed on the plot.

A similar tendency was revealed for dielectric and electrical characteristics (Figures 4 and 5). The defrosting of hybrid networks leads to the increase of the mobility of charge carriers, which, in our case, are sodium cations Na^+^ and protons H^+^ (in some cases). The rise of mobility of the charge carriers has a stepped view in accordance to transitional defrosting of structural formations of both hybrid networks. Figure 6 shows the dependencies of electrical losses *M*″ on the reactivity *R* of the organic component of OIS.

It is obvious that the relaxation maxima near temperatures −90°C, −50°C and 50°C correspond to relaxation processes of low-molecular-weight product, hybrid network MDI/SS and hybrid network PIC/SS, respectively. In addition, two relaxation processes were found in the middle temperature range, which concerns the defrosting of water molecules and interphase polarization (Maxwell-Wagner-Sillars polarization). The temperatures of the relaxation processes are noted in Table 4.

**Table 4:** DRS studies: temperatures of the relaxation processes

**Compositions**			**Relaxation temperatures (*ω*** **= 1 Hz)**		
**Reactivity (*R*)**	**MDI (%)**	**PIC (%)**	***T***_**r0 **_**(°C)**	***T***_**r1 **_**(°C)**	***T***_**r2 **_**(°C)**
0.04	100	0	−98	−60	-
0.06	90	10	−96	−54	-
0.1	80	20	−91	−52	41
0.14	65	35	−90	−51	59
0.18	50	50	−89	−56	70
0.22	35	65	−88	−65	98
0.26	20	80	−87	−76	108

Compositions and glass transition temperatures of OIS obtained from DRS investigations at frequency *ω* = 1 Hz, depending on the reactivity *R* of the organic component of OIS.

## Discussion

It is obvious from the DSC, DMTA and DRS studies that the general properties as well as the structure of OIS depend on the reactivity of the organic component that was regulated by the variation of the ratio between MDI and PIC in the organic component in the reactive mixture during polymerization, dimensions of dominant hybrid network and mineral phase. The rise of the reactivity *R* of the organic component of OIS by increasing the content of the isocyanate-containing modifier PIC leads to the formation of more rigid, thermostable, less conductive and polarisable OIS. The essential changes of these characteristics occurred in the middle range of the reactivity *R* of the organic component, while for low and high values of reactivity *R*, they were more or less invariable. In OIS with low values of reactivity *R*, the major part of the organic component was macrodiisocyanate; thus, the hybrid organic-inorganic network MDI/SS was the dominant structure, and the general properties of OIS were prevalently defined by the properties of this hybrid network. Hybrid network PIC/SS was in the form of domains in matrix of hybrid network MDI/SS. Otherwise, the hybrid network PIC/SS dominated in OIS with high values of reactivity *R*, and the general properties of OIS were prevalently defined by the properties of this network. Also, as it was shown in [13], the OIS with low values of *R* and, correspondingly, the dominant hybrid network MDI/SS contain nano-scale inclusions of the SS mineral phase, whereas the OIS with high values of *R* and, correspondingly, the dominant hybrid network PIC/SS contain micro-dimensional inclusions of the SS mineral phase. The nano-scale inclusions of the SS mineral phase in OIS with the dominant lowly cross-linked network MDI/SS have much highly developed specific active surface with higher number of charge carriers as compared to the micro-dimensional inclusions of the SS mineral phase in the OIS with the dominant highly cross-linked network PIC/SS. Such distributive behavior of charge carriers leads to a higher charge transfer and, correspondingly, ionic conductivity in OIS with dominant ionomeric lowly cross-linked network MDI/SS as compared to highly cross-linked network PIC/SS. In OIS with middle values of reactivity *R*, both networks may be dominant, depending on the prevailing product in the organic component. The transition from domination of hybrid network MDI/SS to domination of hybrid network PIC/SS can be pointed near 0.18 of reactivity *R* of the organic component. In accordance to [20], such OIS can be referred to hybrids with covalently connected building blocks and, in some cases, interpenetrating networks. Thus, for understanding the relationships between structure and properties of such hybrid systems, it is evidently necessary to consider both hybrid networks separately.

### Hybrid network MDI/SS

**Figure 7:**
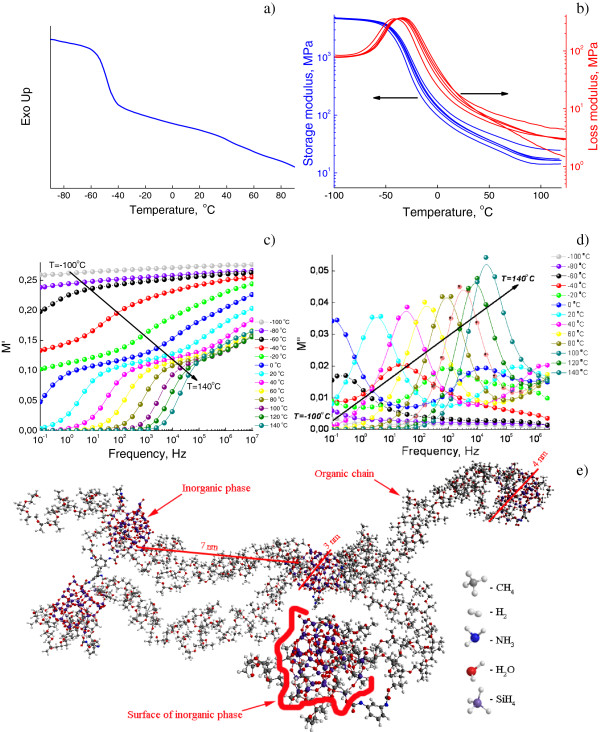
**Spectra and structural model of hybrid network MDI/SS in OIS.** DSC **(a)**, DMTA **(b)** and DRS **(c**, **d)** spectra and structural model **(e)** of the hybrid network MDI/SS in OIS with *R* = 0.06.

Hybrid organic-inorganic network MDI/SS was formed in reactions of high-molecular-weight macrodiisocyanate with two end-functional NCO groups and sodium silicate. This network with low reactivity *R* of organic component and glass transition temperature near −50°C (Figure 7) is characterized by high molecular mobility (Figure 7a), elasticity (Figure 7b), number and mobility of charge carriers (Figure 7c,d) and, correspondingly, relatively high values of permittivity and conductivity. Long organic chains are connected to mineral phase with two end-functional groups (Figure 7e); thus, a weakly cross-linked structure is formed that has bulk adsorbed water.

### Hybrid network PIC/SS

**Figure 8:**
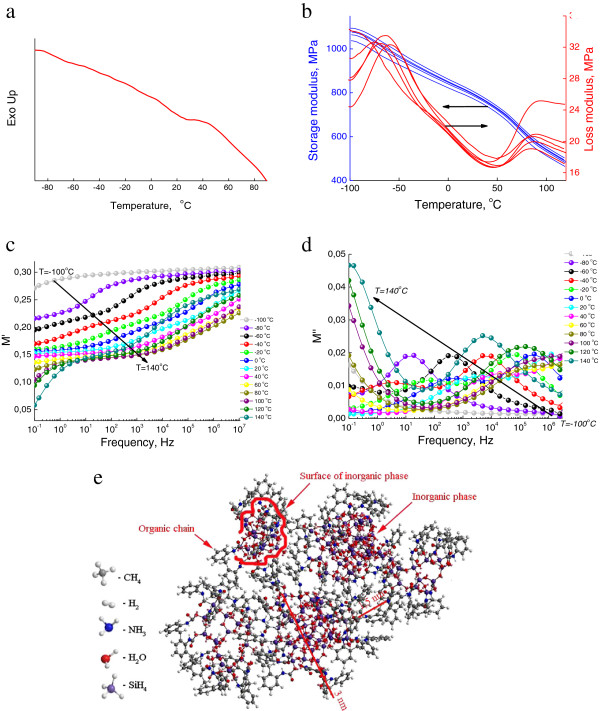
**Spectra and structural model of hybrid network PIC/SS.** DSC **(a)**, DMTA **(b)** and DRS **(c**, **d)** frequency spectra and structural model **(e)** of hybrid network PIC/SS in OIS with *R* = 0.22.

Hybrid organic-inorganic network PIC/SS was obtained in reactions of low-molecular-weight isocyanate-containing modifier poly(isocyanate) with *R* = 0.32 and sodium silicate. This hybrid network is rigid (Figure 8b) with glass transition temperature near 70°C (Figure 8a). The structure of this hybrid network is highly cross-linked with low molecular mobility (Figure 8e), due to the short length of organic chains and high reactivity of organic component. Short organic chains with *R* = 0.32 create continuous layer on the surface of mineral phase. The permittivity and conductivity are low (Figure 8c,d) because of the impossibility of charge transport through such highly cross-linked structure.

## Conclusions

Hybrid organic-inorganic polymer nanosystems (OIS) were obtained in reactions of the organic component that was a mixture of two products: macrodiisocyanate (MDI) and isocyanate-containing modifier poly(isocyanate) (PIC) with inorganic component, namely, water solution of sodium silicate (SS) that exists in a form of oligomer. Changing the reactivity of the organic component from *R* = 0.04 (pure MDI) to *R* = 0.32 (pure PIC), the structure and properties of OIS were varied.

The structure of OIS existed in a form of hybrids with covalently connected building blocks and interpenetrating networks, namely, the lowly cross-linked network as a result of reactions of high-molecular-weight MDI with SS and highly cross-linked network that was created in the reactions of low-molecular-weight PIC with SS. Depending on the MDI/PIC ratio, one of the networks was prevailing and created continuous structure with domains of the second network. The properties of the two types of hybrid networks were strongly different. The general properties of OIS were prevalently defined by the properties of the dominant hybrid network.

## Abbreviations

DMTA: dynamic mechanical thermal analysis; DRS: dielectric relaxation spectroscopy; DSC: differential scanning calorimetry; MDI: macrodiisocyanate; OIS: hybrid organic-inorganic polymer nanosystems; PIC: poly(isocyanate); R: reactivity of organic component; SS: sodium silicate.

## Competing interests

The authors declare that they have no competing interests.

## Authors’ contributions

MI performed all the DSC measurements, structure simulation and wrote the manuscript. YM and GB provided valuable discussions and helped with the results analysis. GS, EL and SI contributed in the analysis and interpretation of the data and compared the results to the structural models. EN assisted in the DRS investigations and analysis of the DRS results. OG helped with the operation of DMTA and interpretation of the DMTA data. All authors read and approved the final manuscript.

## Authors’ information

MI is Doctor in Polymer Physics, senior staff scientist of the Institute of Macromolecular Chemistry of the National Academy of Sciences of Ukraine (NAS of Ukraine), deputy director of the Centre for Thermophysical Investigations and Analysis of the NAS of Ukraine, and assistant coordinator of the Central and East European Polymer Network in Ukraine. YM is a Professor, Dr. Hab. in Polymer Physics and Ph.D. degree holder in Macromolecular Chemistry. He is also a leading staff scientist of the Institute of Macromolecular Chemistry of the NAS of Ukraine and the director of the Centre for Thermophysical Investigations and Analysis of the NAS of Ukraine. GB is Dr. Hab. in Physics and the Director of Research CNRS, Université de Lyon, Université Lyon 1, Ingénierie des Matériaux Polymères, UMR CNRS 5223, IMP@LYON1. GS is a Professor, and Dr. Hab. in Polymer Chemistry, Université de Lyon, Université Lyon 1, Ingénierie des Matériaux Polymères, UMR CNRS 5223, IMP@LYON1. EN is (at the time of the investigations) Doctor in Polymer Physics, Université de Lyon, Université Lyon 1, Ingénierie des Matériaux Polymères, UMR CNRS 5223, IMP@LYON1. OG is an engineer at the Université de Lyon, Université Lyon 1, Ingénierie des Matériaux Polymères, UMR CNRS 5223, IMP@LYON1. EL is a Professor, Dr. Hab in Macromolecular Chemistry, the director of the Institute of Macromolecular Chemistry of the NAS of Ukraine. SI is (at the time of the investigations) Doctor in Macromolecular Chemistry and a leading staff scientist of the Institute of Macromolecular Chemistry of the NAS of Ukraine.
